# Heart rate variability in critical care medicine: a systematic review

**DOI:** 10.1186/s40635-017-0146-1

**Published:** 2017-07-12

**Authors:** Shamir N. Karmali, Alberto Sciusco, Shaun M. May, Gareth L. Ackland

**Affiliations:** Translational Medicine & Therapeutics, William Harvey Research Institute, Queen Mary University of London, John Vane Science Centre, Charterhouse Square, London, EC1M 6BQ UK

**Keywords:** Autonomic, Heart rate variability, Human, Systematic review

## Abstract

**Background:**

Heart rate variability (HRV) has been used to assess cardiac autonomic activity in critically ill patients, driven by translational and biomarker research agendas. Several clinical and technical factors can interfere with the measurement and/or interpretation of HRV. We systematically evaluated how HRV parameters are acquired/processed in critical care medicine.

**Methods:**

PubMed, MEDLINE, EMBASE and the Cochrane Central Register of Controlled Trials (1996–2016) were searched for cohort or case–control clinical studies of adult (>18 years) critically ill patients using heart variability analysis. Duplicate independent review and data abstraction. Study quality was assessed using two independent approaches: Newcastle–Ottowa scale and Downs and Black instrument. Conduct of studies was assessed in three categories: (1) study design and objectives, (2) procedures for measurement, processing and reporting of HRV, and (3) reporting of relevant confounding factors.

**Results:**

Our search identified 31/271 eligible studies that enrolled 2090 critically ill patients. A minority of studies (15; 48%) reported both frequency and time domain HRV data, with non-normally distributed, wide ranges of values that were indistinguishable from other (non-critically ill) disease states. Significant heterogeneity in HRV measurement protocols was observed between studies; lack of adjustment for various confounders known to affect cardiac autonomic regulation was common. Comparator groups were often omitted (*n* = 12; 39%). This precluded meaningful meta-analysis.

**Conclusions:**

Marked differences in methodology prevent meaningful comparisons of HRV parameters between studies. A standardised set of consensus criteria relevant to critical care medicine are required to exploit advances in translational autonomic physiology.

**Electronic supplementary material:**

The online version of this article (doi:10.1186/s40635-017-0146-1) contains supplementary material, which is available to authorized users.

## Background

Autonomic changes are evident from the onset of acute pathology requiring critical care. Cardiac autonomic function can be derived by analysing variability between heart beats to yield time domain and frequency domain (power spectral density) measures that reflect autonomic modulation of cardiac frequency [1, 2]. Heart rate variability (HRV) appears to contribute diagnostic and prognostic value in various cardiometabolic conditions associated with subclinical autonomic dysfunction that predispose to critical illness including hypertension, coronary artery disease, heart failure and diabetes [3–7]. Similarly, HRV has been proposed to serve as a potential diagnostic and prognostic tool in critically ill patients [8].

However, HRV measures in critically ill patients are fraught with potential problems. [9] Although population norms for HRV parameters have been reported in healthy populations [10], the impact of multiple physiological, procedural and technical factors in critically ill patients has not undergone systematic scrutiny in critical care medicine [11]. Moreover, the validity of HRV as a tool to interrogate autonomic function is increasingly under physiological scrutiny [12, 13], since a strong correlation between HRV and morbidity/mortality appears to be largely attributable to incident heart rate. In addition, recording technique, clinical context and adjustment for incident heart rate are key factors to consider when interpreting the translational relevance of HRV in critically ill patients.

Here, we sought to systematically evaluate the methodology and design of HRV studies in critical care medicine. We focused on whether recommended standards for measurement and reporting have been employed [14, 15], with the aim of identifying areas to refine in future HRV experimental design in critical care medicine.

## Methods

### Identification of studies

A literature review was performed based on the Preferred Reporting Items for Systematic reviews and Meta-Analyses (PRISMA) guidelines for systematic reviews [16]. The summary of the search strategy employed is shown in Additional file 1.

We searched the electronic databases PubMed, EMBASE, MEDLINE and the Cochrane Central Register of Controlled Clinical Trials for articles investigating HRV measurement in intensive care patients. Inclusion criteria were full-text studies written in English involving adult patients, published after 1996 (following published guidelines) and reporting traditional time and frequency domain parameters [15]. Studies which reported newer analysis techniques of HRV (e.g. entropy analysis) were excluded, as we focussed on those reporting measures in line with recent European guidance [17]. The following Medical Subject Headings (MESH) were used to identify pertinent articles: “Heart rate variability OR HRV AND Sepsis”, “Heart rate variability OR HRV AND multiple organ dysfunction OR MODS”, “Heart rate variability OR HRV AND critical illness”, “Heart rate variability OR HRV AND intensive care OR ICU”. The last search took place on 9 November 2016. We screened articles by title search and abstract review. Relevant articles were analysed for eligibility, and further articles were identified from reference lists. Articles were excluded based on the following criteria: experimental studies, incorrect target population (adult; >18 years old), medical field other than intensive care, not original research, topic not within scope or traditional HRV parameters not reported.

### Data extraction

Data was extracted by two independent reviewers (S.K and A.S) and recorded into a standardised excel sheet recording: author, year of publication, study design, number of subjects, mean patient age, proportion of male subjects, risk stratification score, comparator groups, study aim and outcome, study design, protocol for measurement, processing, analysis and reporting of HRV parameters, adjustment and reporting of confounding factors and quality assessment. We identified the following clinical confounding factors: age, gender, average heart rate, average respiratory rate, co-morbidities, drugs, sedative drugs, vasoactive drugs, enteral nutrition and mechanical ventilation. Full details of the impact on HRV of these parameters are provided in Additional file 1. For reporting and analysis purposes, we selected the most commonly used time and frequency domain HRV parameters [15].

### Risk of bias and study quality assessment

The quality of studies was assessed by two assessors independently (SK, SM) using two established tools (Newcastle–Ottowa scale, Downs and Black Instrument). The Downs and Black instrument is recommended by the Cochrane Collaboration for use in non-randomised and observational studies (Additional file 1) [18, 19]. Inter-observer reliability evaluating quality within five domains: reporting, external validity, bias, confounding and power. Five questions were omitted because they are designed for interventional trials. The version which we employed in this study therefore has a maximum score of 22. Differences between reviewers were resolved by panel consensus opinion following further review of the article(s) in question by the senior author.

## Results

### Study selection

We identified 238 studies which underwent screening by title search and abstract review. From these, 31 articles involving 2090 patients (including controls) met the inclusion criteria for assessing the role of HRV in critically ill patients [20–53]. Two articles analysed the same cohort of patients [34, 37].

### Study characteristics

**Fig. 1:**
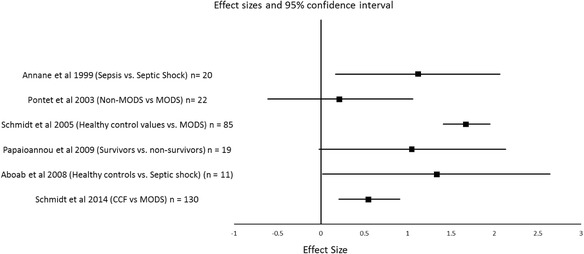
Forest plot of individual effect sizes (Cohen’s *d*) across six studies investigating the relationship between LF/HF ratio and disease severity and mortality

Demographic and clinical data, including comparator groups are summarised in Table 1. All articles reported cohort or case–control studies. The average age of patients was 60 ± 7 years. The majority of studies (22/31; 71%) explored the association between HRV measures, morbidity and mortality. Key clinical findings from these studies are summarised in Table 2. Due to significant differences in trial design, methodology, confounding, non-standardised comparator groups and inconsistent reporting of summary data, a meta-analysis could not be performed. However, there was consistency between studies in their findings that LF/HF ratio was inversely associated with increased disease severity or mortality. For illustrative purposes, the individual effect sizes across six studies reporting mean and standard deviation data looking at disease severity and mortality using the most commonly reported HRV parameter (LF/HF ratio) are shown (Fig. 1).

**Table 1:** Demographics and study design of studies

Reference number. author	Year	Study design	Study populations (± comparator group)	Patients (*n*)	Age (mean ± SD or mean [range])	Male (%)
20. Annane	1999	Case–control	Sepsis (healthy controls)	26	Septic shock 52 ± 14 Sepsis 54 ± 17 Control 43 ± 11	65
21. Korach	2001	Cohort	Sepsis	41	50 [20–90]	44
22. Barnaby	2002	Cohort	Sepsis	15	59 [39–85]	–
23. Pontet	2003	Case–control	Sepsis + MODS (Sepsis − MODS)	22	MODS 59.5 ± 17.8 Non-MODS 60 ± 10.4	64
24.Shen	2003	Cohort	Weaning	24	Successful wean 76 ± 12.9 Unsuccessful wean 69.8 ± 17.8	42
25. Schmidt	2005	Cohort	MODS (literature values)	85	60.4 ± 14	62
26. Papaioannou	2006	Cohort	MODS	53	63.02 ± 14.68	58
44. Bourgault	2006	Cohort	Mixed aetiology	18	60 [33–82]	72
45. Chen	2007	Cohort	Sepsis	81	67 [30–84]	41
50. Passariello	2007	Case–control	Ischaemic sudden death	40	Sudden death 66 ± 8 Pathology matched controls 68 ± 8	
46. Chen	2008	Cohort	Sepsis	132	67 [27–86]	47
47. Aboab	2008	Case–control	Sepsis ± adrenal insufficiency (healthy controls)	81	Septic shock and adrenal failure 55 ± 16 Septic shock 58 ± 19 Healthy controls (not provided)	36
27. Nogueira	2008	Cohort	Sepsis	31	Survivors 44.9 ± 5.9 Non-survivors 55.6 ± 4.63	74
28. Papaioannou	2009	Cohort	Sepsis (Sepsis SOFA <10)	45	57.8	–
51. Tiainen	2009	Cohort	Out of hospital cardiac arrest	70	Hypothermia 60 (23–75) Normothermia 59 (18–75)	86
29. Schmidt	2010	Case–control^a^	MODS	178	61.1 ± 13.2	67
30. Kasaoka	2010	Cohort	SIRS	10	53 ± 15	70
31. Chen	2012	Case–control	Sepsis and out of hospital cardiac arrest (Non-severe sepsis and healthy controls)	210	Out of hospital cardiac arrest 68 ± 10 Severe sepsis and mechanical ventilation 66 ± 8 Severe sepsis 68 ± 7 Sepsis 67 ± 6 Healthy 66 ± 6	55
32.Gomez Duque	2012	Cohort	Sepsis (literature values)	100	55 [18–88]	42
33. Brown	2013	Cohort	Sepsis	48	57 [40–63]	46
34. Green	2013	Cohort	MODS	33	56.5 ± 15.9	61
35.Wieske	2013	Cohort	ICU acquired weakness	83	ICU acquired weakness 60 ± 13 No ICU acquired weakness 59 ± 16	60
36. Wieske	2013	Cohort	Mixed aetiology (healthy controls)	32	Patients 54 ± 15 Healthy control 36 ± 2	70
37. Bradley	2013	Cohort	Mixed aetiology	33	56.5 ± 15.9	61
38. Huang	2014	Cohort	Mixed aetiology	101	Successful 65 ± 18 Unsuccessful 71 ± 16	65
39. Zhang	2014	Cohort	SIRS/MODS (non-MODS)	41	47 [34–59]	54
40. Schmidt	2014	Case–control^a^	CCF and MODS (literature values)	130	CCF 63 ± 10.1 MODS 62.8 ± 10.2	63
52. Tang	2014	Case–control	Stroke	227	AF stroke 74 ± 12 Non-AF stroke 62 ± 15 Age/sex-matched controls 61 ± 10	40
41. Zaal	2015	Case–control	ICU delirium (no delirium)	25	ICU delirium 67 ± 12 No ICU delirium 57 ± 16	72
42. Hammash	2015	Cohort	Weaning	35	53.3 ± 14.6	66
53. Nagaraj	2016	Case series^a^	Not specified	40	56.3 ± 16.8	62.5

Reference for each paper is shown before first author (first column)

*CCF* congestive cardiac failure, *MODS* multiple organ dysfunction syndrome, *SIRS* systemic inflammatory response syndrome, *SOFA* sequential organ failure assessment

^a^Retrospective analysis

**Table 2:** Study objectives and key findings

Author	Year	Study objectives	Key findings
Annane	1999	Compare HRV between sepsis, septic shock and healthy volunteers	TP, LF, LFnu, LF/HF lower in septic shock vs sepsis
Korach	2001	Effects of sepsis, age, sedation, catecholamines and illness severity on sympathovagal balance (LF/HF)	LF/HF ratio <1.5 was associated with sepsis and mortality
Barnaby	2002	Assess if HRV can predict sepsis severity	Negative correlation between LFnu, LF/HF and SOFA score
Pontet	2003	Assess if HRV can predict MODS in sepsis	Low LF and RMSSD associated with MODS
Shen	2003	Assess changes in cardiac autonomic activity during weaning from mechanical ventilation	HF, LF and TP decreased in unsuccessful group during spontaneous breathing trial
Schmidt	2005	Effects of MODS, age, sedation, catecholamines, mechanical ventilation on HRV Assess if HRV can predict mortality in MODS	Time and frequency domain reduced in MODS HRV indices affected by mechanical ventilation but not age, sedation or catecholamines LnVLF associated with 28-day survival.
Papaioannou	2006	Assess if HRV associated with disease severity and mortality	LF/HF ratio negatively correlated with SOFA score
Bourgault	2006	Effects of endotracheal suction on HRV	No significant differences found in HRV indices between closed or open suctioning
Chen	2007	Assess if HRV can predict sepsis severity	Septic shock associated lower LF, LFnu, LF/HF, and higher RMSSD, HF, HFnu
Passariello	2007	Assess if HRV can predict ischaemic sudden cardiac death	SDNN decreases shortly before ischaemic sudden death
Chen	2008	Assess if HRV can predict 28-day mortality	Low SDNN, TP, VLF, LF and LF/HF associated with increased 28-day mortality
Aboab	2008	Assess effect of steroids on HRV in patients with sepsis	LF, LFnu, LF/HF lower in septic shock. Corticosteroids helped increase LFnu values in adrenal insufficiency group.
Nogueira	2008	Assess relationship between HRV, markers of myocardial damage and free fatty acids in sepsis	Low LF, HF and LF/HF associated with mortality
Papaioannou	2009	Assess relationship between HRV and biomarkers of inflammation (CRP, IL-6, IL-10) in patients with sepsis	There is a negative correlation between LFnu, LF/HF and CRP, IL-6, IL-10, SOFA score
Tiainen	2009	Assess if HRV changes (and has prognostic ability) with therapeutic cooling of resuscitated cardiac arrest patients	Higher SDNN, SDANN, TP, LF, HF in the first 48 h of cooling. SDNN >100 ms predicts better neurological outcome
Schmidt	2010	To assess if ACE-I therapy affects short (28-day) and long (365-day) mortality in patients with MODS	ACE-I associated with preserved VLF, LF, HF, TP and survival (28-day and 365-day)
Kasaoka	2010	To trial a real-time HRV measurement and analysis system	LF, HF and LF/HF higher in patients spontaneously breathing compared to mechanical ventilation
Chen	2012	To compare HRV between post-resuscitation cardiac arrest patients and patients with severe sepsis	No significant differences in HRV indices between OOHCA and Severe Sepsis patients Low LF, LFnu, LF/HF associated with mortality
Gomez Duque	2012	To investigate the incidence of cardiovascular adverse events in patients with sepsis	Deceased patients demonstrated lower SDNN than survivors
Brown	2013	Assess if HRV can predict vasopressor dependence at 24 h in sepsis	Traditional HRV indices not associated with vasopressor requirement after controlling for HR
Green	2013	Association of HRV and illness severity in MODS	Low LFnu and LF/HF associated with increased MODS
Wieske	2013	Relationship between autonomic dysfunction (HRV) and ICU-acquired weakness	Artefacts, mechanical ventilation, sedation, catecholamines and heart rate all associated with TP % artefacts were associated with TP and LF/HF No association between HRV and ICU-acquired weakness
Wieske	2013	Compare different autonomic function tests in critically unwell patients (CFT, SWT and HRV)	Only HRV tests associated with SOFA score
Bradley	2013	Impact of sedation and sedation interruptions on HRV	SDNN, RMSSD and HF all increased during sedation interruption (more pronounced in less unwell patients)
Huang	2014	Assess if HRV associated with weaning success or failure	Reduction in TP during SBT associated with failure
Tang	2014	Assess if HRV predicts outcome in ICU stroke patients	Traditional HRV indices were unable to predict outcome
Zhang	2014	Asses if HRV can predict infected pancreatic necrosis or MODS in patients with severe acute pancreatitis	Low LFnu, LF/HF and high HFnu associated with increased MODS and mortality
Schmidt	2014	Assess relationship between HRV and illness severity in CCF and MODS	MODS patients demonstrated lower HRV indices in all parameters compared to CCF patients.
Zaal	2015	To assess if HRV is abnormal in patients with ICU delirium.	No association between HRV and delirium found
Hammash	2015	Assess relationship between HRV and incidence of dysrhythmias during weaning	LF was higher during spontaneous breathing than during controlled mechanical ventilation.
Nagaraj	2016	Assess if sedation levels can be classified by HRV algorithms	Algorithms using composite measures of HRV may discriminate between levels of sedation in ICU patients

*ACE-I* angiotensin-converting enzyme inhibitor, *CCF* congestive cardiac failure, *CFT* cold face test, *CRP* C-reactive protein, *HF* high frequency, *HFnu* high frequency normalised unit, *HRV* heart rate variability, *IL-6* interleukin 6, *IL-10* interleukin 10, *LF* low frequency, *LFnu* low frequency normalised unit, *MODS* multiple organ dysfunction, *RMSSD* root mean square of successive differences, *SOFA* sequential organ failure assessment, *SBT* spontaneous breathing trial, *SWT* skin wrinkle test, *TP* total power, *VLF* very low frequency, *LnVLF* natural logarithm of very low frequency

### Quality of studies

No studies reported Strengthening the Reporting of Observational Studies in Epidemiology (STROBE) guidelines. Two studies analysed data retrospectively. A minority of studies (*n* = 5; 16%) used individualised HRV data—i.e. patients serving as their own control, prior to an intervention. More than one third of studies (*n* = 13; 42%) did not describe any comparator group. The remainder of studies used non-age matched healthy volunteers, non-critically ill patients with established cardiovascular disease or HRV values derived from the literature. External validity (as adjudged by the Down and Black assessment tool) was poor, with the majority of studies achieving a score of 1.

### Risk of bias assessment

We found recurring potential sources of bias in study design, with 19 (61%) studies failing to report whether HRV data analysers were masked to the patient condition/outcome (Additional file 1). Only one study performed a power calculation [41].

### Data acquisition and preparation

Details on short-term recordings, including source of heart rate periods [54, 55], duration of recordings, epochs used for analysis and patient position [56] were variable or not reported. Fourteen (45%) studies did not describe the sampling frequency of recordings; four (13%) studies used sampling rates below the recommended 250 Hz [15].

ECG recording in the critically ill population is frequently contaminated by electrical and physiological artefacts. Thus, detailing methods to detect artefact (manual or automated) and its management (segment selection, deletion or interpolation) is important for data interpretation [57]. Fourteen (45%) studies reported automated and/or manual editing of the raw ECG to remove artefact by replacing the missing data with cubic spline or linear interpolation methods. In keeping with guidelines, the majority of studies used interpolation methods as opposed to deletion of abnormal beats to avoid a loss of information [15].

### HRV analysis

Measurement protocols, processing and reporting of HRV data are summarised in Table 3.

**Table 3:** Procedures for measurement, processing and reporting of HRV

Author	Year	Recording protocol (duration/position/time)	Monitor	Sampling frequency (Hz)	Management of artefact	Data presented
Annane	1999	5 min/–/–	PRV	500	Interpolation	TP, LF, HF, LF/HF, Lfnu, Hfnu
Korach	2001	30 min supine/0800–1200	ECG	5	Interpolation	Lfnu, Hfnu, LF/HF
Barnaby	2002	5 min/–/–	ECG	–	Interpolation	TP, LF, HF, Lfnu, Hfnu, LF/HF
Pontet	2003	10 min/supine/2100–2300	ECG	>500	Interpolation	SDNN, RMSSD, LF, HF, Lfnu, Hfnu, LF/HF
Shen	2003	90 min/semi recumbent/1000–1400	ECG	–	Interpolation	TP, LnLF, LnHF, Lfnu, Hfnu, LF/HF
Schmidt	2005	24 hours	ECG	256	Interpolation	SDNN, SDANN, RMSSD, pNN50, VLF, LF, HF, LF/HF
Papaioannou	2006	10 min/supine/morning	ECG	250	Segment selection	LF/HF
Bourgault	2006	20 min/–/day and night	ECG	1000	–	LF, HF, LF/HF, TP
Chen	2007	10 min/supine/day and night	ECG	–	Interpolation	RMSSD, TP, LF, HF, Lfnu, Hfnu, LF/HF
Passariello	2007	24 h	ECG	–	–	SDNN, SDANN, pNN50, RMSSD
Chen	2008	10 min/supine/day and night	ECG	–	Interpolation	SDNN, RMSSD, TP, LF, HF, Lfnu, Hfnu, LF/HF
Aboab	2008	5 min/supine/–	PRV	–	Interpolation	TP, Lfnu, Hfnu, LF/HF
Nogueira	2008	30 min/supine/morning	ECG	–	–	LF, HF, LF/HF
Papaioannou	2009	10 min/–/–	ECG	250	Segment selection	SDNN, Lfnu, Hfnu, LF/HF
Tiainen	2009	24 h	ECG	–	–	SDNN, SDANN, TP, LF, HF,
Schmidt	2010	24 h	ECG	256	Interpolation	LnTP, LnVLF, LnHF, LnLF, LF/HF
Kasaoka	2010	5 min/supine/–	ECG	–	–	LnLF, LnHF, LF/HF
Chen	2012	10 min/supine/day and night	ECG	–	Interpolation	SDNN, TP, VLF, LF, HF, Hfnu, Lfnu, LF/HF
Gomez Duque	2012	24 h	ECG	–	–	SDNN, PNN50
Brown	2013	6 h/–/–	ECG	500	Deletion	SDNN, pNN50, Lfnu, Hfnu, LF/HF
Green	2013	24 h	ECG	125	Deletion	SDNN, RMSSD, Lfnu, Hfnu, LF/HF
Wieske	2013	5 min/–/–	ECG	250	Interpolation	HR, TP, LF/HF
Wieske	2013	5 min/supine/–	ECG	250	Deletion	LF, HF, Lfnu, Hfnu, LF/HF
Bradley	2013	24 h	ECG	125	Deletion	SDNN, RMSSD, LF, HF, LF/HF
Huang	2014	5 min/semi-recumbent/0800–1200	ECG	–	–	LnTP, LnVLF, Hfnu, Lfnu, LF/HF
Tang	2014	60 min/–/–	ECH	512	–	SDNN, RMSSD, LF, HF, LF/HF
Zhang	2014	5 min/–/0900–1100	ECG	–	Deletion	SDNN, RMSSD, TP, VLF, LF, HF, Lfnu, Hfnu, LF/HF
Schmidt	2014	24 h	ECG	256	Interpolation	SDNN, SDANN, SDNNi, RMSSD, pNN50, VLF, LF, HF, LnLF, LnHF, LF/HF
Zaal	2015	15 min/supine, 0800–1700	ECG	500	Segment selection	LnLF, LnHF, Hfnu, LF/HF
Hammash	2015	24 h	ECG	–	Interpolation	VLF, HF, LF
Nagaraj	2016	24 h (5 min epochs)	ECG	240	Thresholding	SDNN, RMSSD, VLF, LF, HF, LF/HF, LFnu, HFnu

*ECG* electrocardiogram, *HF* high frequency, *HFnu* high frequency normalised unit, *LF* low frequency, *LFnu* low frequency normalised unit, *Ln* natural logarithm, *pNN50* percentage of normal–normal intervals >50 ms, *PRV* pulse rate variability, *RMSSD* root mean square of successive differences, *SDANN* standard deviation of average normal–normal intervals, *SDNN* standard deviation of normal–normal intervals, *TP* total power

A minority of studies (14; 45%) reported both frequency and time domain data (Table 3). A minority of studies (9; 29%) reported frequency data in normalised units together with absolute values, in keeping with established recommendations. Summary values for commonly reported HRV parameters revealed a wide range of non-normally distributed data for each (Additional file 1: Table S3). Reporting and/or adjustment for heart rate and respiratory rate, which dramatically alter both high and low frequency spectral components [58] was inconsistent between studies. A small majority of studies (17; 55%) reported average heart rate, whilst a minority (6; 19%) adjusted for, or reported, respiratory rate during data acquisition.

### Pharmacologic and clinical interventions

Studies varied in their exclusion criteria and reporting of potential confounding factors including age, gender, body mass index [59], common comorbidities [60–63], drug therapy [64–68] and/or ICU interventions (Tables 4 and 5). Exclusion criteria used and comorbidities/drugs are summarised in Additional file 1. A minority of studies (12; 39%) excluded patients with chronic comorbidities that are commonly associated with autonomic dysfunction. Reporting of drugs that directly affect autonomic function was highly variable across studies. A majority of studies (25; 81%) did not detail drug therapy. Around 22% studies did not report the use of mechanical ventilation, and more than 25% failed to report whether sedation and/or vasoactive drugs were used at the time of HRV recordings.

**Table 4:** Reporting of potential clinical confounders

Author	Year	Comorbidities	Drugs	Mechanical ventilation (% patients)	Sedation (% patients)	Catecholamines (% patients)	Feeding	HR/RR reported
Annane	1999	Excluded	–	100%	0%	0%	–	HR/RR
Korach	2001	–	–	41.5%.	19.5%	12.20%	–	–
Barnaby	2002	–	–	0%	–	0%	–	HR/RR
Pontet	2003	Excluded	Excluded	38.5%	–	17.90%	–	HR
Shen	2003	+	+	100%	0%	0%	–	HR/RR
Schmidt	2005	–	–	71%	61%	62%	–	–
Papaioannou	2006	+	–	–	+	–	–	–
Bourgault	2006	Excluded	Excluded	100%	33%	0%	–	HR
Chen	2007	Excluded/+	–	–	–	0%	–	HR/RR
Passriello	2007	+	+	–	–	–	–	HR
Chen	2008	+	–	0%	–	–	–	HR
Aboab	2008	Excluded	–	100%	80.9%	100%	–	HR
Nogueira	2008	Excluded	–	100%	–	100%	–	RR
Papaioannou	2009	–	Excluded	100%	100%	–	–	–
Tiainen	2009	+	–	100%	100%	87%	–	HR
Schmidt 0	2010	+	–	88%	89%	74%	–	–
Kasaoka 1	2010	–	–	100%	100%	–	–	–
Chen	2012	+	–	OHCA 100%, SS + MV 100%, SS 0%, S 0%	OHCA 81, SS + MV 63%, SS 59%, S 0%	OHCA 100%, SS + MV 9%, SS 18.8%, S 0%	–	HR
Gomez Duque	2012	Excluded/+	–	–	–	72%	–	–
Brown	2013	–	–	–	–	63%	–	HR
Green	2013	–	–	90.90%	+	78.80%	–	HR
Wieske	2013	Excluded/+	+	+	+	+	–	HR
Wieske	2013	Excluded/+	–	100%	–	–	–	–
Bradley	2013	–	–	+	+	+	–	HR
Huang	2014	Excluded/+	Excluded/+	100%	–	–	–	RR
Zhang	2014	–	–	–	–	12%	–	–
Schmidt	2014	–	+	89.2%	72.3%	72.3%	–	HR
Tang	2014	+	+	–	–	–	–	HR
Zaal	2015	Excluded	Excluded	60%	20%	0%	–	–
Hammash	2015	Excluded/+	–	100%	–	–	–	–
Nagaraj	2016	–	–	100%	100%	–	–	HR

Excluded refers to specific comorbidities or drugs were part of exclusion criteria of study

*HR* heart rate, *RR* respiratory rate, *+* reported but proportion of patients not provided, *–* not reported

**Table 5:** Reporting of potential confounders

Author	Year	Comorbidities	Drugs	Mechanical ventilation (% patients)	Sedation (% patients)	Catecholamines (% patients)	Feeding	HR/RR reported
Annane [17]	1999	Excluded	–	100%	0%	0%	–	HR/RR
Korach [18]	2001	–	–	41.5%.	19.5%	12.20%	–	–
Barnaby [19]	2002	–	–	0%	–	0%	–	HR/RR
Pontet [20]	2003	Excluded	Excluded	38.5%	–	17.90%	–	HR
Shen [21]	2003	+	+	100%	0%	0%	–	HR/RR
Schmidt [22]	2005	–	–	71%	61%	62%	–	–
Papaioannou [23]	2006	+	–	–	+	–	–	–
Bourgault [24]	2006	Excluded	Excluded	100%	33%	0%	–	HR
Chen [25]	2007	Excluded/+	–	–	–	0%	–	HR/RR
Passriello	2007	+	+	–	–	–	–	HR
Chen [26]	2008	+	–	0%	–	–	–	HR
Aboab [27]	2008	Excluded	–	100%	80.9%	100%	–	HR
Nogueira [28]	2008	Excluded	–	100%	–	100%	–	RR
Papaioannou [29]	2009	–	Excluded	100%	100%	–	–	–
Tiainen	2009	+	–	100%	100%	87%	–	HR
Schmidt [30]	2010	+	–	88%	89%	74%	–	–
Kasaoka [31]	2010	–	–	100%	100%	–	–	–
Chen [32]	2012	+	–	OHCA 100%, SS + MV 100%, SS 0%, S 0%	OHCA 81, SS + MV 63%, SS 59%, S 0%	OHCA 100%, SS + MV 9%, SS 18.8%, S 0%	–	HR
Gomez Duque [33]	2012	Excluded/+	–	–	–	72%	–	–
Brown [34]	2013	–	–	–	–	63%	–	HR
Green [35]	2013	–	–	90.90%	+	78.80%	–	HR
Wieske [36]	2013	Excluded/+	+	+	+	+	–	HR
Wieske [37]	2013	Excluded/+	–	100%	–	–	–	–
Bradley [38]	2013	–	–	+	+	+	–	HR
Huang [39]	2014	Excluded/+	Excluded/+	100%	–	–	–	RR
Zhang [40]	2014	–	–	–	–	12%	–	–
Schmidt [41]	2014	–	+	89.2%	72.3%	72.3%	–	HR
Tang	2014	+	+	–	–	–	–	HR
Zaal [42]	2015	Excluded	Excluded	60%	20%	0%	–	–
Hammash [43]	2015	Excluded/+	–	100%	–	–	–	–
Nagaraj	2016	–	–	100%	100%	–	–	HR

Excluded refers to specific comorbidities or drugs were part of exclusion criteria of study

*HR* heart rate, *RR* respiratory rate, + reported but proportion of patients not provided, – not reported

## Discussion

This review is the first to systematically explore how HRV analyses are undertaken and/or reported in critically ill patients. Despite a wealth of laboratory and translational data suggesting that HRV may offer diagnostic and prognostic utility, significant heterogeneity in methodology between HRV articles precluded comparisons across studies and meta-analysis. Our review identifies several areas that require greater scrutiny in future, highlighting the need to develop consensus guidelines that are relevant and tailor-made for the challenges faced by researchers in critical care medicine.

Well-recognised technical, physiologic and clinical factors impact on the measurement, and interpretation of HRV [69, 70]. We found highly variable practice in three key technical areas. Low sampling rates (<250 Hz) impair the precise detection of the R wave fiducial point in the ECG waveform, which consequently affects the power spectrum [15]. This is particularly relevant for studies that derived R–R intervals from arterial pressure waveform analysis [20, 47], since non-neural respiratory influences (e.g. changes in ventricular mechanics) differentially affect mechanical pulse waves and electrical R waves [55]. Manual inspection of the raw ECG to identify artefact is preferred to automated methods to avoid the introduction of false frequency components into the power spectrum [57]. The variable (or unstated) masking of HRV analysers to clinical data also introduces potential significant bias.

From a physiologic perspective, reporting and/or adjustment for heart rate and respiratory rate was inconsistent between studies, with heart rate frequently not reported. Across species with highly variable heart rates, HRV appears to be largely attributable to incident heart rate. If heart rate is not taken into account, erroneous conclusions regarding HRV are likely since differences may merely reflect lower heart rate [12]. This is particularly of relevance to hemodynamically unstable critically ill patients, in whom heart rate may rapidly change. Similarly, increases in respiratory frequency and tidal volume affect both high and low frequency spectral components [58]. Hence, standardised criteria for ventilatory and heart rate reporting are required for the interpretation of HRV data between studies (and hence, potentially, meta-analysis).

From a clinical perspective, HRV parameters are influenced strongly by age, gender, functional capacity and chronic comorbidities. Whilst all studies estimated severity of illness, the most frequently employed—Acute Physiology and Chronic Health Evaluation II (APACHE-II)—are limited in capturing information about chronic comorbid disease that are over-represented in the critical care medicine population. For example, diabetes mellitus, a common condition associated with cardiac autonomic neuropathy, is not captured by this type of assessment [60]. Typically, chronic conditions at the severe end of the disease spectrum are included (e.g. APACHE-II score only includes severe heart failure (≥NYHA class 3). However, HRV parameters have been found to be abnormal in early cases of chronic disease, including preserved ejection fraction, coronary artery disease, chronic kidney disease and hypertension [60–63]. Although some studies have considered these factors, serial measures or dynamic autonomic challenges offer a potentially more insightful and individualised approach to assessing HRV. Novel HRV parameters that can be captured within the first few minutes of critical illness, such as deceleration capacity of heart rate [71], may mitigate the need for refining the use of more traditional time and frequency domain measures. For mechanistic studies investigating whether changes in autonomic parameters correlate with, or precede, pathologic events, targeting clinical scenarios where multiple, complementary baseline autonomic measures [72, 73] can be made before critical illness develops may be optimal [74]. Studies where basal autonomic function can be captured, including elective surgery [73–76] and oncologic sepsis [48, 49], may provide particularly powerful mechanistic insights since autonomic changes can be individualised and referenced to pre-insult normal, or pre-existing, dysfunction. Several studies have highlighted that HRV values in critical care medicine are similar to those found in common cardiovascular pathologic conditions [74, 75, 77]; this highlights the need for individualised patient data in order to rule out that autonomic dysfunction is not a precursor of critical illness, rather than merely a biomarker.

Commonly used anti-arrhythmic drugs, anti-hypertensive drugs, statins, metformin and inhaled bronchodilators have all been associated with changes in HRV parameters [60–63]. However, the lack of reporting on medications that critically ill patients received reduces the mechanistic insight afforded by this approach, particularly given the strong correlation between HRV and morbidity/mortality appears to be largely attributable to incident heart rate. Similarly, the majority of studies in this review failed to consistently report on the use of common critical care interventions. This may explain why conflicting conclusions over how variety of features of critical illness may affect HRV. Continuous enteral or parenteral nutrition are both associated with a reduction in time domain HRV measures indicative of parasympathetic cardiac modulation [67]. However, we did not find any studies that reported on the feeding or fasting status of patients. Although a significant limitation of our study was the lack of primary source data, in any event, we could not identify a single common HRV parameter measured in all studies that enabled comparison. A further limitation is that we did not consider newer nonlinear and multiscale approaches, since very few studies incorporating these analyses have been undertaken. These approaches are also likely to be affected by the same factors that influence traditional HRV parameters [78]. Thus, in a clinical setting, further work is required to establish whether these newer approaches reduce the impact of several confounding factors we have identified in this review.

## Conclusions

Heart rate and derived heart rate variability offers a non-invasive, inexpensive tool that may add mechanistic insights to our understanding of critical illness and also assist clinical care. However, the current interpretation of generalizable and clinically relevant values to aid clinical decisions/research is hampered by a non-standardised methodologic approach and lack of adjustment for important confounding factors. For critical care medicine to exploit recent advances in translational autonomic physiology, further high-quality prospective HRV studies underpinned by the development of consensus reporting standards relevant for critical care medicine are needed.

## Additional file

Additional file 1: Clinical confounding factors. (DOCX 34 kb)

## Abbreviations

HRV: Heart rate variability
APACHE-II: Acute Physiology and Chronic Health Evaluation II
NYHA: New York Heart Association
ECG: Electrocardiogram
MODS: Multiple organ dysfunction syndrome
