# Supplementary material for: Heart rate variability in critical care medicine: a systematic review
Source: Intensive Care Med Exp. 2017 Jul 12;5:33. doi: 10.1186/s40635-017-0146-1 (PMC5507939; doi:10.1186/s40635-017-0146-1)
Supplement: Additional file 1: — Clinical confounding factors. (DOCX 34 kb) [file 40635_2017_146_MOESM1_ESM.docx]

**Supplementary Digital content 1. Clinical confounding factors**

| **Confounding Factor** | **Publication** | | |
| --- | --- | --- | --- |
|  | **Author** | **Year** | **Journal** |
| Age | Antelmi | 2004 | Am J Cardiology |
| Sex | Antelmi | 2004 | Am J Cardiology |
| Functional capacity | Antelmi | 2004 | Am J Cardiology |
| Body position | Montano | 1994 | Circulation |
| Heart rate (mathematical argument) | Sacha | 2014 | Ann Noninvasive Electrocardiol |
| Heart rate (biophysical argument) | Monfredi | 2014 | Hypertension |
| Respiratory rate | Brown | 1993 | J Applied Physiol |
| Mechanical ventilation | Wieske | 2013 | Intensive Care Med |
| Sedation | Wieske | 2013 | Intensive Care Med |
| Vasoactive drugs | Wieske | 2013 | Intensive Care Med |
| Artificial feeding (enteral and parenteral) | Gale | 2012 | Shock |
| Anti-arrythmic drugs (B-blockers, anticholinergics) | Akselrod | 1981 | Science |
| ACE-I | Akselrod | 1981 | Science |
| Antihypertensives | Pavithran | 2010 | Clinical and Experimental Pharmacology and Physiology |
| B-agonists | Cekici | 2009 | Br J Clin Pharmacol |
| Statins | Vrtovec | 2005 | Journal of Cardiac Failure |
| Metformin | Manzella | 2004 | Hypertension |
| Coronary artery disease (risk factors) | Thayer | 2010 | Int J Cardiol |
| Myocardial infarction | Buccelletti | 2009 | Eur Rev Med Pharmacol Sci |
| Hypertension | Singh | 1998 | Hypertension |
| Heart failure | Nolan | 1998 | Circulation |
| CKD | Drawz | 2013 | Am J Nephrol |
| Smoking | Dinas | 2013 | Int J Cardiol |
| COPD | Roque | 2014 | Int Arch Med |
| Diabetes Mellitus | Stuckey | 2013 | Crit Rev Biomed End |
| Cerebrovascular disease/stroke | De Raedt | 2015 | J Neurol Sci |

**Supplementary digital content 2: Quality of studies assessed using the Downs and Black Instrument.**

Quality was evaluated within five domains: Reporting, External Validity, Bias, Confounding and Power. Five questions (Q4, Q8, Q19, Q23 and Q24) were omitted because they are designed for interventional trials.

| **Author** | **Year** | **Down and Black Quality Assessment** | | | | | |
| --- | --- | --- | --- | --- | --- | --- | --- |
|  |  | **Reporting**  **(Max 8)** | **External Validity**  **(Max 3)** | **Bias**  **(Max 6)** | **Confounding**  **(Max 4)** | **Power**  **(Max 1)** | **Total**  **(Max 22)** |
| Annane | 1999 | 8 | 1 | 3 | 2 | 0 | 14 |
| Korach | 2001 | 8 | 2 | 4 | 3 | 0 | 17 |
| Barnaby | 2002 | 7 | 2 | 3 | 2 | 0 | 14 |
| Pontet | 2003 | 8 | 1 | 5 | 2 | 0 | 16 |
| Shen | 2003 | 9 | 1 | 4 | 4 | 0 | 18 |
| Schmidt | 2005 | 8 | 3 | 5 | 3 | 0 | 19 |
| Papaioannou | 2006 | 7 | 1 | 4 | 3 | 0 | 15 |
| Bourgault | 2006 | 7 | 1 | 3 | 2 | 0 | 13 |
| Chen | 2007 | 8 | 3 | 5 | 4 | 0 | 20 |
| Passariello | 2007 | 7 | 1 | 2 | 3 | 0 | 13 |
| Chen | 2008 | 7 | 3 | 5 | 3 | 0 | 18 |
| Aboab | 2008 | 7 | 1 | 3 | 2 | 0 | 13 |
| Nogueira | 2008 | 8 | 3 | 4 | 4 | 0 | 19 |
| Papaioannou | 2009 | 7 | 1 | 4 | 3 | 0 | 15 |
| Tiainen | 2009 | 8 | 2 | 5 | 4 | 0 | 19 |
| Schmidt | 2010 | 7 | 1 | 5 | 2 | 0 | 15 |
| Kasaoka | 2010 | 8 | 1 | 3 | 1 | 0 | 13 |
| Chen | 2012 | 7 | 1 | 3 | 2 | 0 | 13 |
| Gomez Duque | 2012 | 7 | 1 | 3 | 2 | 0 | 13 |
| Brown | 2013 | 7 | 3 | 3 | 3 | 0 | 16 |
| Green | 2013 | 7 | 1 | 3 | 2 | 0 | 13 |
| Wieske | 2013 | 7 | 3 | 4 | 3 | 0 | 17 |
| Wieske | 2013 | 7 | 3 | 3 | 3 | 0 | 16 |
| Bradley | 2013 | 8 | 1 | 4 | 4 | 0 | 17 |
| Huang | 2014 | 8 | 3 | 4 | 3 | 0 | 18 |
| Zhang | 2014 | 8 | 3 | 3 | 4 | 0 | 18 |
| Tang | 2014 | 8 | 1 | 2 | 3 | 0 | 14 |
| Schmidt | 2014 | 7 | 1 | 4 | 0 | 0 | 12 |
| Zaal | 2015 | 7 | 1 | 3 | 2 | 0 | 13 |
| Hammash | 2015 | 8 | 1 | 3 | 3 | 0 | 15 |
| Nagaraj | 2016 | 5 | 1 | 2 | 0 | 0 | 7 |

**Supplementary Table 3. Summary of reported HRV frequency data.**

*Data from studies by Bradley and Green involved the same cohort of patients. Non-standardised units (NSU) are where studies reported values in units different from ms^2^. Studies not reporting these data are marked with x.

| **Author** | **Year** | **Groups** | **Acuity Score** | **TP** | **VLF** | **LF** | **HF** | **LF/HF** | **HFnu** | **LFnu** |
| --- | --- | --- | --- | --- | --- | --- | --- | --- | --- | --- |
| Annane | 1999 | Septic shock | SAPS II: 58±23 | NSU | NSU | NSU | NSU | NSU | NSU | NSU |
|  |  | Sepsis | SAPS II: 41 ± 16 | NSU | NSU | NSU | NSU | NSU | NSU | NSU |
| Korach | 2001 | sepsis | SAPS II: 22 (6-53) | x | x | x | x | 1.58 | 4.22 | 3.65 |
| Barnaby | 2002 | sepsis | A-II:14 (5-27) | NSU | NSU | NSU | NSU | NSU | NSU | NSU |
| Pontet | 2003 | Sepsis (MODS) | A-II:16±7 | x | x | 11.3 | 14.8 | 2.3 | 48.3 | 51.7 |
|  |  | Sepsis (non-MODS) | A-II:13±6 | x | x | 108.8 | 43.7 | 2.9 | 31.2 | 68.8 |
| Shen | 2003 | Successful wean | A-II:21±6 | x | x | x | x | 1.43 | 19.3 | 80.7 |
|  |  | Unsuccessful wean | A-II:21±4 | x | x | x | x | 1.42 | 19.4 | 80.6 |
| Schmidt | 2005 | MODS | A-II:28±8 | x | 191.3 | 129.3 | 112.3 | 1.1 | x | x |
| Papaioannou | 2006 | Survivors | SOFA: 7±3 | x |  | x | x | 3.2 | x | x |
|  |  | Non-survivors |  | x | x | x | x | 2.19 | x | x |
| Bourgault | 2006 | Pre-open suction | Not reported | 112.52 | x | 15.97 | 24.75 | 0.76 | x | x |
| Chen | 2007 | Sepsis | MEDS:7±4 | **32.9** | **x** | **7.6** | **1.2** | **4.5** | **4.1** | **18.9** |
|  |  | Septic shock | MEDS:7 ±5 | **24.5** | **x** | **3.5** | **4.8** | **0.7** | **23** | **13** |
| Passariello | 2007 | Sudden death | Not reported | x | x | x | x | x | x | X |
|  |  | Pathology matched controls | Not reported | x | x | x | x | x | x | x |
| Chen | 2008 | Survivors | MEDS 8±5 | **30.6** | **x** | **6.7** | **1.6** | **3.1** | **6.4** | **18** |
|  |  | Non-survivors | MEDS 16±6 | **8.8** | **x** | **1.2** | **0.6** | **0.8** | **24.8** | **18.9** |
| Aboab | 2008 | Septic shock +adrenal failure | SOFA 8±3 | NSU | NSU | NSU | NSU | NSU | NSU | NSU |
|  |  | Septic shock | SOFA 9±3 | NSU | NSU | NSU | NSU | NSU | NSU | NSU |
| Nogueira | 2008 | Survivor | A-II 27±3 | x | x | 50 | 10 | 0.65 | x | x |
|  |  | Non-survivor | A-II 26±2 | x | x | 320 | 120 | 0.4 | x | x |
| Papaioannou | 2009 | Sepsis | A-II 18±6 | x | x | x | x | x | 1.52 | 1.76 |
| Tiainen | 2009 | Hypothermia | Not reported | 13.2 | x | 9.9 | 8.2 | x | x | x |
|  |  | Normothermia | Not reported | 8.3 | x | 5.7 | 4.8 | x | x | x |
| Schmidt | 2010 | MODS (ACE-I) | A-II 30±8 | x | x | x | x | 1.3 | x | x |
|  |  | MODS (No ACE-I) |  | x | x | x | x | 1.3 | x | x |
| Kasaoka | 2010 | SIRS (MV) | SOFA 7±3 | x | x | x | x | 2.5 | x | x |
|  |  | SIRS (SB) |  | x | x | x | x | 7.2 | x | x |
| Chen | 2012 | OOHCA | SAPS II:61 (56-72) | **21.3** | **9.8** | **5.1** | **3.9** | **1** | **49.7** | **50.3** |
|  |  | Severe sepsis + MV | SAPS II:60 (54-65) | **24.6** | **13.6** | **4.7** | **4.4** | **1.1** | **48.7** | **51.3** |
|  |  | Severe sepsis | SAPS II:59 (48-61) | **19.3** | **11.4** | **3.7** | **4** | **0.8** | **54.1** | **45.9** |
| Gomez Duque | 2012 | Sepsis | A-II: 17±10 | x | x | x | x | x | x | X |
| Brown | 2013 | Sepsis | A-II: 19 [15-29] | x | x | x | x | 1.74 | 37 | 63 |
| Green | 2013 | Mild MODS | A-II 23±7 | x | x | x | x | **2.29** | **7.97** | **18.3** |
|  |  | Moderate MODS |  | x | x | x | x | **2.29** | **5.98** | **9.23** |
|  |  | Severe MODS |  | x | x | x | x | **1.73** | **5.67** | **8.58** |
| Wieske | 2013 | ICU weakness | A-IV: 83±27 | **20** | x | x | x | **1** | x | x |
|  |  | ICU no weakness | A-IV: 66±22 | **2** | x | x | x | **2** | x | x |
| Wieske | 2013 | 1 | A-II: 15 [6-25] | **21.3** | x | **11.1** | **10.5** | **x** | **34.9** | **66.7** |
| Bradley | 2013 | Mild MODS (sedated) | A-II 23±7 | x | x | 14.7 | 10.2 | 3.24 | x | x |
|  |  | Mild MODS (sedation hold) |  | x | x | 22.9 | 34.2 | 2.88 | x | x |
|  |  | Moderate MODS (sedated) |  | x | x | 6.68 | 4.28 | 2.48 | x | x |
|  |  | Moderate MODS (sedation hold) |  | x | x | 8.6 | 7.31 | 3.22 | x | x |
|  |  | Severe MODS (sedated) |  | x | x | 6.48 | 4.07 | 2.02 | x | x |
|  |  | Severe MODS (sedation hold) |  | x | x | 9.83 | 5.48 | 2.78 | x | x |
| Huang | 2014 | Pre-SBT | A-II 17±6 | x | x | x | x | 1 | 45 | 38 |
|  |  | During SBT |  | x | x | x | x | 1 | 47 | 38 |
| Zhang | 2014 | MODS | A-II: 14 [11-18] | 126.3 | 12.5 | 2.7 | 17.7 | x | 31.1 | 4.5 |
|  |  | Non-MODS |  | 232.8 | 72.4 | 60.7 | 10.8 | x | 5.6 | 42 |
| Schmidt | 2014 | CCF | A-II: 7±3 | x | 491.8 | 285.6 | 223.9 | 2 | x | x |
|  |  | MODS | A-II:31±7 | x | 140 | 119.6 | 101.1 | 1.1 | x | x |
| Tang | 2014 | Non-AF stroke | MRS: <2 | x | x | 418.9 | 338.4 | 2.1 | x | x |
|  |  | Non AF stroke | MRS: >3 | x | x | 378.4 | 569.8 | 1.9 | x | x |
| Zaal | 2015 | ICU Delirium | A-IV: 74 [62-85] | x | x | x | x | **0.45** | **73** | x |
|  |  | No ICU delirium | A-IV: 74 [37-82] | x | x | x | x | **0.85** | **62** | x |
| Hammash | 2015 | 1 | A-IV 70±25 | x | 79.4 | 82.9 | 78.8 | x | x | x |
| Nagaraj | 2016 | A | Not reported | x | 102.58 | 108.77 | 80.52 | 1.35 | 21.77 | 29.4 |
|  |  | B |  | x | 83.02 | 90.92 | 98.86 | .94 | 28.98 | 27.2 |
|  |  | C |  | x | 85.38 | 100.48 | 133.36 | .75 | 33.48 | 25.22 |
|  |  | D |  | x | 71.43 | 84.18 | 97.77 | .86 | 30.57 | 26.32 |
| MEDIAN | | |  | 21 | 79 | 15 | 11 | 1.5 | 30 | 28 |
| 25^th^-75^th^ centile | | |  | 16-32 | 14-103 | 7-100 | 5-98 | 1-2 | 8-46 | 18-51 |

ACE-I (Angiotensin Converting Enzyme Inhibitor), AF (atrial fibrillation), A-II-IV; APACHE score (Acute physiology and chronic health evaluation score), MEDS (mortality in emergency department sepsis score), MODS (multi-organ dysfunction score), MRS (modified Rankin Scale), MV (mechanical ventilation), OOHCA (out of hospital cardiac arrest), SAPS (Simplified acute physiology score), SB (spontaneous breathing), SBT (spontaneous breathing trial), SOFA (sequential organ failure assessment score).
